# Exploring Extended White Blood Cell Parameters for the Evaluation of Sepsis among Patients Admitted to Intensive Care Units

**DOI:** 10.3390/diagnostics13142445

**Published:** 2023-07-21

**Authors:** Sook Fong Ho, Swee Jin Tan, Mohd Zulfakar Mazlan, Salfarina Iberahim, Ying Xian Lee, Rosline Hassan

**Affiliations:** 1 Department of Haematology, School of Medical Sciences, Universiti Sains Malaysia, Kubang Kerian 16150, Kelantan, Malaysia; hsf_sookfong@yahoo.com (S.F.H.); salfarina@usm.my (S.I.); 2 Transfusion Medicine Unit, Hospital Universiti Sains Malaysia, Kubang Kerian 16150, Kelantan, Malaysia; 3 Sysmex Asia Pacific Pte Ltd., Singapore 528735, Singapore; lee.yingxian@sysmex.com.my; 4 Department of Anaesthesiology, School of Medical Sciences, Universiti Sains Malaysia, Kubang Kerian 16150, Kelantan, Malaysia; zulfakar@usm.my

**Keywords:** sepsis, Sequential Organ Failure Assessment (SOFA), haematology, extended inflammatory parameters, cell population data

## Abstract

Sepsis is a major cause of mortality and morbidity in intensive care units. This case–control study aimed to investigate the haematology cell population data and extended inflammatory parameters for sepsis management. The study included three groups of patients: sepsis, non-sepsis, and healthy controls. Patients suspected of having sepsis underwent a Sequential Organ Failure Assessment (SOFA) evaluation and had blood drawn for blood cultures, complete peripheral blood counts (CBC), and measurements of various markers such as C-reactive protein (CRP), procalcitonin (PCT), and interleukin-6 (IL-6). We observed significant changes in numerous CBC parameters and extended inflammation parameters (EIPs), in addition to significant biochemical analysis markers CRP and IL-6 in sepsis cohorts. Multiple logistic regression analyses showed that combining different CBC parameters and EIPs were effective to profile these patients. Two different models have been developed using white blood cell counts and their extended parameters. Our findings indicate that the absolute counts of white blood cells, and the EIPs which reflect their activation states, are important for the prediction and assessment of sepsis, as the body responds to an insult that triggers an immune response. In an emergency situation, having timely updates on patient conditions becomes crucial for guiding the management process. Identifying trends in these specific patient groups will aid early diagnosis, complementing clinical signs and symptoms, especially as CBC is the most commonly ordered test in a diagnostic workup.

## 1. Introduction

Sepsis is a life-threatening condition caused by the body’s response to an infection that results in organ dysfunction [1,2]. It is a serious complication and a leading cause of mortality in intensive care units (ICUs), with a mortality rate of around 26% [3]. The Society of Critical Care Medicine and the European Society of Intensive Care Medicine have proposed a definition of sepsis based on life-threatening organ dysfunction caused by a dysregulated host response to infection, assessed using the Sequential Organ Failure Assessment (SOFA) scoring system [1,4]. The SOFA evaluates six systems: the nervous, cardiovascular, renal, haematology, pulmonary, and gastrointestinal systems [5]. Clinical diagnosis of sepsis requires meeting the criteria of organ dysfunction, defined as an increase of 2 points or more in the SOFA score [6]. Patients meeting the SOFA score are predicted to have a mortality of ≥10%. Early diagnosis and appropriate management of sepsis can help reduce mortality and morbidity.

Various testing assays are available to detect the presence of sepsis and monitor its progression [7]. To overcome the limitations of blood culture in detecting sepsis, especially in culture-negative sepsis, some newer molecular techniques such as polymerase chain reaction (PCR) and next-generation sequencing (NGS) have been shown to be most promising methods to improve sensitivity and reducing the time required for diagnosis [8,9,10]. Other tests, such as procalcitonin (PCT) and C-reactive protein (CRP), are widely used as biomarkers of sepsis [11], but their specificity and sensitivity may vary depending on the type of infection and individual patient characteristics [12]. Additionally, point-of-care tests such as lactate measurements [13] and bedside ultrasonography [14] can aid in the early identification and management of sepsis. However, it is important to note that no single test is sufficient for diagnosing sepsis, and clinical judgment and a comprehensive evaluation of patient history and symptoms are also essential in performing a timely and accurate diagnosis [15,16]. Blood cell parameter testing is a quick and easy diagnostic tool that healthcare professionals use to identify and monitor a wide range of diseases. With just a small sample of blood, laboratory specialists can perform various tests to detect markers of diseases such as infections [17], cancer [18], autoimmune disorders [19], and metabolic abnormalities [20]. Moreover, blood cell parameter testing has become increasingly efficient with the advancement of technology, enabling faster and more accurate results [21,22,23]. Overall, the ease and convenience of blood cell parameters make them an indispensable tool for healthcare professionals in diagnosing and managing a variety of diseases. White blood cell research parameters from haematology analysers have been actively studied in the early detection of sepsis, as demonstrated by Urrechaga et al. [24].

This case–control study evaluating the diagnostic performance of extended blood parameters from a haematology analyser in predicting and assessment of sepsis among patients in the ICU is of utmost importance. Specifically, extended white cell parameters, including the Neut-GI, Neut-RI, AS-Lymph, RE-Lymph, and RE MONO, are tested. These are indirect indicators of activated white blood cells derived during routine CBC testing [25]. Sepsis is a life-threatening condition that necessitates prompt diagnosis and treatment to enhance patient outcomes [26]. However, diagnosing sepsis can be challenging due to its shared symptoms with other conditions and negative blood culture [27]. This study can potentially identify more precise and efficient diagnostic methods, enabling earlier identification and management of sepsis. We hypothesize that specific research parameters within each complete blood count (CBC) can show trends that are indicative of the immune response in septic conditions. By systematically analysing these trends, we aimed to build useful models that can support their clinical use in combination with other assays. Therefore, this study has the potential to make a significant impact on patient care and improve the overall management of sepsis.

## 2. Materials and Methods

### 2.1. Study Design

The case–control study was conducted at the Hospital Universiti Sains Malaysia (HUSM) in the Intensive Care Unit (ICU) and the Transfusion Medicine Unit. The study was approved by The Human Research Ethics Committee (JEPeM) of Universiti Sains Malaysia (USM) (USM/JEPeM/21010131) from June 2020 to June 2022. Patients recruited for this study were selected based on specific inclusion criteria. In the septic group, potential patients in the ICU were initially identified with a Sequential Organ Failure Assessment (SOFA) score greater than or equal to 2. Confirmation of sepsis was subsequently made by their attending physician using the Sepsis 3.0 criteria [28], taking into account additional clinical signs and symptoms. This approach ensured the early collection of blood samples for analysis. For the non-septic group in the ICU, patients with a baseline measurement of SOFA less than 2 were initially selected. Throughout their stay in the ICU, these patients were monitored and confirmed to be non-septic. The healthy control group consisted of regular blood donors to the hospital who had normal complete blood count (CBC) values within the healthy reference intervals. By employing these specific inclusion criteria, our study aimed to encompass a diverse range of patients with varying septic and non-septic conditions, while establishing an appropriate baseline with healthy controls. Exclusion criteria were malignancy, chemotherapy, pregnancy, autoimmune disease, neutropenia, steroid therapy, patient refusal for active management, expired patients, and incomplete documentation. All of the patients and healthy volunteers or their proxies provided written informed consent. The analysis included 30 patients with sepsis, 23 patients without sepsis, and 30 healthy control volunteers, after filtering cases that were non-conformal. The inclusion criteria were stringent, ensuring the study’s credibility and the accuracy of the results obtained.

### 2.2. Sample Collection and Analysis

In total, 16.5 mL of whole blood was obtained upon patients’ admission to the intensive care unit. For this purpose, 3 mL peripheral blood samples were taken using Becton Dickinson (BD, Franklin Lakes, NJ, USA) vacutainer K_2_EDTA and an XN-1000 haematology analyzer (Sysmex Corp., Kobe, Japan) was used to perform complete blood cell counts, while the FinecareTM FIA system (Model No: FS-113/FS-205) was used to perform Finecare^TM^ CRP (Wondfo, Guangzhou, China) and Procalcitonin Rapid Quantitative Test. All samples were processed within an hour of sample collection. Subsequently, 3.5 ml whole blood samples were collected in the BD vacutainer SSTTM tube and allowed to clot for about 30 min and then centrifuged at 3500 rpm for 4 min to obtain the serum. The Elabscience^®^ Human Interleukin 6 (IL-6) ELISA kit (Elabscience^®^ Houston, TX, USA), which operates on the Sandwich-ELISA principle with Sunrise-Basic Tecan, was used to analyse the serum sample for IL-6. Moreover, 10 mL of peripheral blood was obtained in BD BACTECTM Plus Aerobic/F medium, which was incubated for a minimum of 5 days in the BD BACTEC 9240 system. The identification of bacteria was performed using Gram stain processes, followed by manual testing with multiple rapid tests and biochemical tests or by automated identification with VITEK^®^ 2 (Biomerieux, Marcy l’Etoile, France) once a positive vial from BAC-TEC 9240 was obtained. Another 6.5 ml of whole blood was obtained on Day 3 and Day 5 for the purpose of serial monitoring of the CBC, EIPs, PCT, CRP, and IL-6.

### 2.3. Research Cell Population Data Derived from the XN-1000 Haematology Analyzer

The extraction of cell population data (CPD) and advanced clinical parameters were performed manually by exporting sample runs from the information processing unit (IPU) of the XN analyser. In this study, we focused on EIPs which were Neut-GI, Neut-RI, AS Lymph, RE Lymph, and RE MONO for correlation to clinical outcomes. This narrowed the analyses to focus solely on immune responses to an infection that can cause sepsis. Backup of data was performed fortnightly to ensure data are not overwritten due to overcapacity. 

### 2.4. Statistical Analysis

The data obtained were expressed as means with standard deviations (SDs) for numerical variables and frequencies (*n*) with percentages (%) for categorical variables, unless otherwise specified. The descriptive statistics and multiple logistic regression model were computed using Statistical Package for Social Sciences (SPSS) Statistics for Windows (Version 26.0, IBM Corp., Armonk, NY, USA). The model with culture negative cases utilizes parameters related to activated white blood cells to ascertain the instantaneous body’s responses to fight infection. Utilizing all significant parameters, the subsequent analysis aimed to confirm predictive capabilities and highlight possible interventional phases during the monitoring of sepsis conditions. For numerical variables that were more than two groups, one-way analysis of variance (ANOVA) was used for the data comparison. Serial measurements of numeric variables of extended inflammatory parameters (EIPs), which were the Neut-GI, Neut RI, AS Lymph, RE Lymph, and RE MONO among the sepsis cohort from Day 0 to Day 5, were analysed by repeated measures ANOVA. 

Obtaining positive blood culture results is currently a challenging task, and we aimed to investigate whether the differences in haematological parameters can help to further stratify sepsis patients with negative cultures in the ICU. We hypothesized that the haematological parameters could serve as biomarkers to predict patient outcomes, which could guide clinical decision-making. To test our hypothesis, we performed multiple logistic regression analysis on the EIPs. Receiver operating characteristic (ROC) curve analysis was performed, and the best cut-off, sensitivity, and specificity were determined by Stata statistical software (Stata MP 17) of (StataCorp., College Station, TX, USA). The results were considered statistically significant if *p*-value < 0.05.

## 3. Results

### 3.1. Demographics Data

This study analysed a total of 83 individuals, and their demographics are presented in Table 1. Of the participants, 16 (53.1%) were male and 14 (46.7%) were female, with a mean age of 58 years and a standard deviation of 13. The participants were classified into sepsis, non-sepsis, and healthy groups. The non-sepsis group consisted of 14 (60.9%) male and 9 (39.1%) female patients, with a mean age of 49 years and a standard deviation of 16. The healthy control group included 20 (66.7%) males and 10 (33.3%) females, with a mean age of 34 years and a standard deviation of 13. Additionally, among patients with sepsis, only 7 (23%) had positive blood culture results, while 23 (76.7%) had negative blood culture. Among patients with sepsis, 20 (66.7%) were discharged and 10 (33.3%) were deceased. All of the 23 (100%) non-sepsis patients were discharged. 

### 3.2. Blood Investigation Profiles among Study Participants

In this study, we compared laboratory variables and clinical scores among three groups; the results are presented in Table 1. We analysed various haematological parameters and found that the sepsis group had significantly higher levels of TWBC, NEUT#, Neut-RI, IG#, RE-Lymph, AS-Lymph, IPF%, and RE-MONO compared with the non-sepsis and healthy control groups. On the other hand, non-sepsis patients in ICU had higher levels of NEUT-GI, monocyte counts, lymphocyte counts, platelet counts, and Delta-He values compared with sepsis patients.

**Figure 1:**
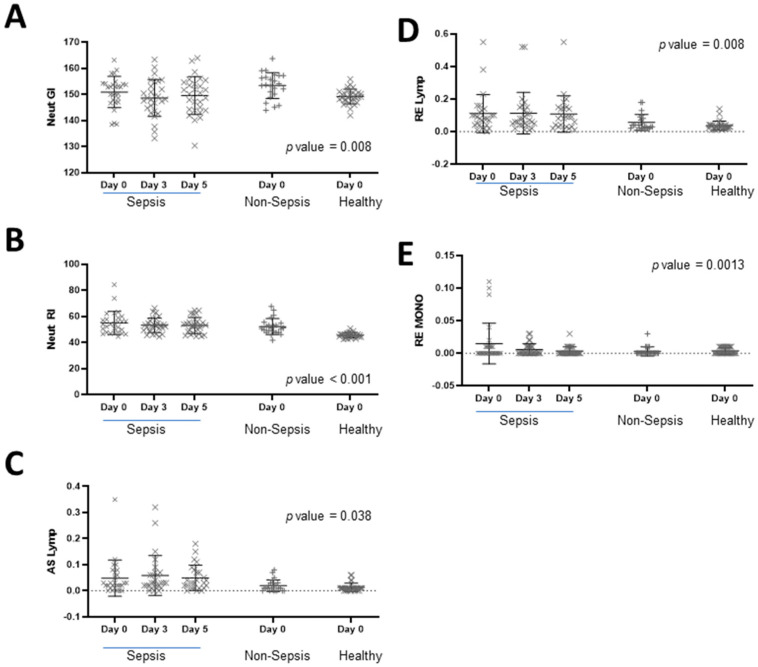
Analysis of extended inflammatory parameters across different subject cohorts. (**A**) Neut-GI; (**B**) Neut-RI; (**C**) RE-MONO; (**D**) AS-Lymph; (**E**) RE-MONO. EIPs measurements were performed across patients with sepsis, non-sepsis cases, and healthy donors. ANOVA results of the day 0 comparison are provided with *p*-values stated in each corresponding graph.

Furthermore, the collective EIPs that indicate activation states of specific white blood cells (as shown in Figure 1) were statistically significant among all three groups, as determined by one-way ANOVA. Interestingly, when we looked at the serial measurements among the sepsis cohort from Days 0 to 5, we did not find any statistically significant differences. The changes in the variables of Neut-GI, Neut-RI, AS-Lymph, RE-Lymph, and RE-MONO in response to antimicrobial therapy on Day 0, Day 3, and Day 5 were expressed using a scatter plot with the mean and standard deviation (Figure 1). 

### 3.3. Haematological Extended Inflammatory Parameters in Culture Negative Septic Patients

**Figure 2:**
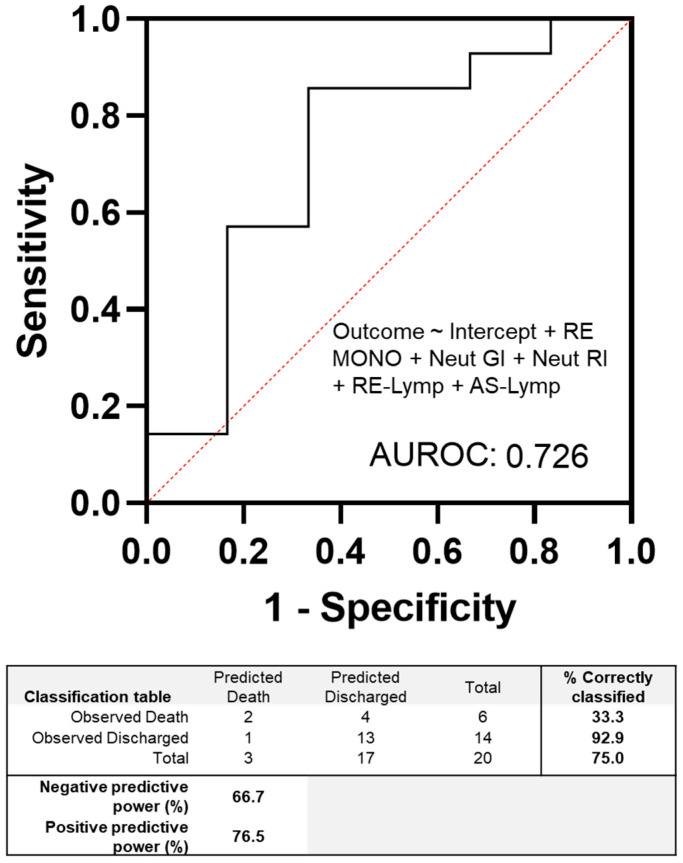
Multiple logistic regression modelling of haematology extended inflammatory parameters on the sepsis cohort with negative blood cultures. The cut-off value was set at 0.5, and the outcome was defined as either patient discharge or death. The table accompanying the figure summarizes the accuracy of the model. One patient result was excluded due to incomplete data on the clinical case report.

The multiple logistic regression analysis was performed on the EIPs shown in Figure 2 to model the outcome of patients with negative cultures, either death or discharge. We used a cut-off of 0.5 to classify patients into these two categories. The limited sample cohort achieved an accuracy of 75% in correctly classifying the outcomes of patients. The positive and negative predictive powers were 76% and 67%, respectively. Interestingly, the results indicated that the EIPs alone correctly identified 92.9% of patients who were discharged using baseline blood measurements. 

### 3.4. Evaluating the Discriminatory Power of Haematological Parameters in Comparison to Classical Sepsis Markers

**Figure 3:**
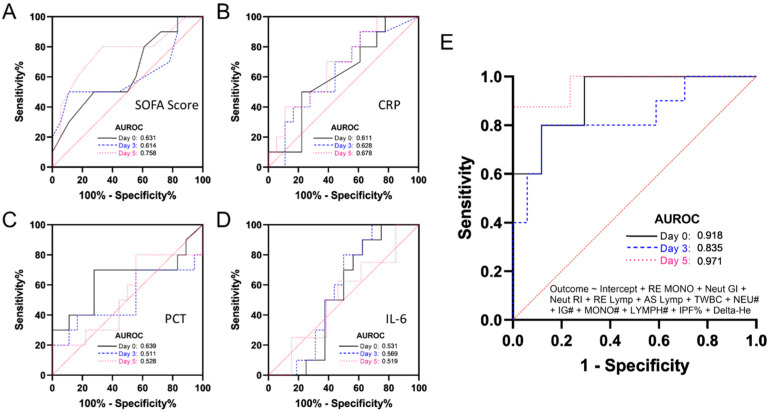
Serial sampling results among the sepsis cohort demonstrated excellent prognostication of patients with worse outcomes. (**A**) Comparison of SOFA scores achieved the highest area under the receiver operating characteristic (AUROC) curve on Day 5 (0.758). (**B**) Receiver operating characteristic (ROC) analysis of CRP data indicated a significant degree of separation among patients who were discharged. (**C**) PCT results for this cohort showed the highest value at the index measurement. (**D**) IL-6 showed marginal effects on the prognostication of sepsis patients. (**E**) The area under receiver operating characteristic curve (AUROC) shows combination of haematological variable of RE-MONO + Neut-GI + Neut-RI + RE-Lymp + AS-Lymp + TWBC + NEU# + IG# + MONO# + LYMPH# + IPF% + Delta-He.

The classic markers of sepsis, such as SOFA, CRP, PCT, and IL-6 on Days 0, 3, and 5 were compared. Serial sampling among the sepsis cohort showed that certain markers could predict worse outcomes in patients, as shown in Figure 3 using ROC analyses. The SOFA scores achieved the highest area under the ROC curve on Day 5 at 0.758, indicating its ability to accurately predict patient outcomes (Figure 3A). Similarly, CRP data showed significant separation among patients who were discharged, suggesting that CRP levels could be a useful marker in predicting recovery, with an increasing AUROC from 0.611 to 0.678, as shown in Figure 3B.

PCT results showed the highest value at Day 0 and were only marginally significant at Day 3 (Figure 3C). IL-6 showed a low value of AUROC among sepsis patients, suggesting that it may not be as useful as other markers in predicting patient outcomes (Figure 3D). On the other hand, haematological variables were modelled using multiple logistic regression and included a number of significant parameters identified earlier, including EIPs, absolute counts for lymphocytes, neutrophils, and monocytes, as well as total white cell counts (TWBCs), IPF%, IG#, and Delta He. These variables demonstrated a strong correlation with patient outcomes, indicating their potential as prognostic markers. The AUROC for Days 0, 3, and 5 were 0.918, 0.835, and 0.971, respectively, in which correlate well with patients’ worst prognosis. 

The area under the receiver operating characteristics curve (AUROC) of the SOFA score for worse outcomes was 0.631 at Day 0, 0.614 at Day 3, and 0.758 at Day 5. (B) The area under the receiver operating characteristics curve (AUROC) of CRP for worse outcomes was 0.611 at Day 0, 0.628 at Day 3, and 0.678 at Day 5. (C) The area under the receiver operating characteristics curve (AUROC) of the PCT for worse outcomes was 0.639 at Day 0, 0.511 at Day 3, and 0.528 at Day 5. (D) The area under the receiver operating characteristics curve (AUROC) of IL-6 for worse outcomes was 0.531 at Day 0, 0.569 at Day 3, and 0.519 at Day 5. (E) The area under the receiver operating characteristic curve (AUROC) of a combination of haematological variables of RE-MONO + Neut-GI + Neut-RI + RE-Lymp + AS-Lymp + TWBC + NEU# + IG# + MONO# + LYMPH# + IPF% + Delta He for worse outcomes was 0.918 at Day 0, 0.835 at Day 3, and 0.971 at Day 5. 

## 4. Discussion

The golden hour for sepsis patients refers to the immediate period after sepsis has been identified and treatment has been initiated. During this critical time, it is essential to provide prompt and aggressive treatment to prevent the condition from worsening and potentially leading to organ failure or death [29]. The goal is to administer antibiotics and fluids within this critical period to improve outcomes and increase the patient’s chances of survival. With sepsis patients, each delay in treatment reduces sepsis survival; we aimed to assess whether a combinatory result from CBC analysis can further aid in this endeavour. CBC testing is integral in existing sepsis management, as it can provide valuable information about the patient’s condition. CBC can reveal abnormalities in white blood cell count and differential, red blood cell count, haemoglobin and haematocrit levels, platelet count, and other parameters. In sepsis, CBC can provide information about the extent of infection and inflammation, as well as the immune response of the patient. Leucocyte alternations, such as leucocytosis and leukopenia, are common among patients with sepsis [30]. CBC can also be used to monitor the response to treatment and to identify any adverse effects of treatment [31]. The rapid identification and treatment of sepsis are essential for improving patient outcomes.

In this study, we explored newer features of automated haematology analysers that use novel gating techniques to assess cell populations related to the activation states of neutrophils, lymphocytes, and monocytes (EIPs). Neut-GI and Neut-RI reflect the reactivity of neutrophils, highlighting the cytoplasmic granulation and metabolic activity, respectively. These can be associated with the early innate immune responses. AS-Lymph and RE-Lymph represent the antibody synthesizing lymphocytes and total reactive lymphocytes, respectively. RE-MONO presents the cluster of reactive monocytes in each specimen [25]. These significant parameters, in association with pathophysiological changes observed in sepsis, are a result from infections impacting the immune system and the body’s response to external insults or infections. These parameters can give more information on the functional activity of different white blood cells in response to sepsis, in addition to the cell count. It is worth mentioning that these parameters have previously been described in the scientific literature as relevant indicators of bacterial infections in the lower respiratory tract [32]. Moreover, subsequent studies have demonstrated their utility as indicators of viral infections and predictors of severity in COVID-19 cases [33,34]. 

This study is novel to assess critical cases that are challenging with blood cell cultures; existing markers do not suffice to meet the needs of these patients. With the EIPs that consist of Neut GI, Neut RI, AS-Lymp, RE-Lymp, and RE MONO, we observed statistical significance among our sepsis group and the control arm of non-sepsis and healthy volunteers. This led to the hypothesis that routine haematological results coupled with EIPs may be a viable test to support clinical decisions in sepsis cases. Among these parameters, Neut RI showed significance for the disease cohort, which reflected an activated neutrophil activity against sepsis. These CBC tests come with no additional burden to patients. Although the frequency of blood draws for CBC testing in the ICU can vary depending on the individual patient’s condition and treatment plan, critically ill patients in the ICU require frequent monitoring of their blood parameters, including CBC, to assess for any changes that may require intervention. This may involve daily or even more frequent blood draws in some cases. Our study provides support for the use of EIP in the stratification of patients in ICUs who may be at risk for worse outcomes. In comparison to other markers such as CRP, repeated monitoring on the same day may not show significant difference, which can help in decision-making for the management of patients. CBC with EIPs has the potential to be a viable test to guide clinical management by serial monitoring, especially in critical conditions in which the patient deteriorates in the episode of sepsis over a short time. 

One interesting aspect of this study is the recognition that sepsis is a complex condition that can trigger diverse immune responses depending on the underlying cause. Therefore, relying on a single blood cell parameter may not be the optimal strategy to accurately characterize these patients. Looking into the whole profile of CBC, especially the white cell count and the EIPs, may give a clearer picture of our immune responses and the activated status of white blood cells in response to infection or sepsis. Indeed, by combining the EIPs to assess blood-culture-negative cases, we observed strong associations with good outcomes, but also identified a subset of patients who unfortunately did not survive. It is possible to adjust the cut-off values to increase the test’s sensitivity for better outcome stratification, but this would require a larger cohort study and a validation group to assess its accuracy. Additionally, incorporating existing known biochemical markers could potentially enhance the discriminatory power of the test and would be a worthwhile avenue for future investigation. Our study provides preliminary data to support future research to target patients with negative blood cultures. 

The diagnosis of sepsis is often subjective and can be challenging due to non-specific symptoms and rapid changes in patients’ conditions, making active monitoring crucial. While the introduction of SOFA scores has helped to standardize the clinical management of sepsis, existing sepsis markers, such as CRP, PCT, and IL-6, may not always accurately predict patient outcomes [16,35]. In our study, we found that the sepsis group had significantly higher levels of TWBC, NEUT#, NEUT-RI, IG#, RE-LYMPH, AS-LYMPH, IPF%, and RE-MONO compared with the non-sepsis and healthy control groups. On the other hand, non-sepsis patients in ICU had higher levels of NEUT-GI, monocyte count, lymphocyte count, platelet count, and Delta-He values compared with sepsis patients. Thus, these parameters have potential for the early diagnosis of sepsis, as these parameters were elevated during sepsis. In this study, we revealed that two culture-negative samples with undesired clinical outcomes were flagged by this modelling using Day 0 CBC results. This finding highlights the potential of haematological parameters to provide valuable prognostic information for patients with negative cultures in the ICU. Furthermore, our model correctly identified approximately 93% of cases for discharge, which could alleviate the burden of emergency personnel.

In addition, we aimed to build useful models that can support their clinical use in combination with other assays by systematically analysing the CBC trends in this study. This can reduce sepsis-related mortality and improve patient outcomes, leading to a more effective and efficient healthcare system. Therefore, this study has the potential to make a significant impact on patient care and improve the overall management of sepsis. In our study, we compared the performance of these markers with serial samplings at Day 0, 3, and 5, and found that the SOFA score was the best predictor of worse outcomes. In all cases, AUROC was not significantly different, except for SOFA score at Day 5, which exhibited much better separation compared with the corresponding index measurement. With the combination of several significant CBC parameters and EIPs (as shown in Figure 3E), the present study demonstrated excellent discriminatory power among sepsis patients who were discharged or had worse outcomes. The AUROC was highest on Day 5, suggesting that a common blood test utilizing the appropriate parameters could be valuable in predicting patient outcomes and guiding treatment decisions. Additionally, we observed that haematological results were much better to stratify patients’ outcomes than all existing markers. Our findings may also assist in adjusting the frequency of blood draws based on the patient’s condition and response to treatment. Ultimately, the treating healthcare team makes the decision regarding the frequency of CBC testing based on the individual patient’s needs.

Consideration should be given to a limitation of the current study, which is the need for a larger validation cohort to confirm the results of the modelling of various haematological parameters. This study serves as a pilot investigation into the use of a simple blood test to provide additional support for the clinical management of sepsis. The model used to assess the activation of different white cells, together with clinical adjudication, is rudimentary and may potentially benefit from a more complex weighted function model. This is particularly important to discern against specific changes within the neutrophil and lymphocyte populations. An example is provided by Nierhaus et al. [36], that optimizing the AUROC considering weighted effects of differential blood cellular parameters. Furthermore, we recognize the value of incorporating cell markers that can be correlated with this indirect measurement of the activation of white cells (EIPs) in future studies. Considering the convenience and rapid results of CBC tests, this study supports clinicians promptly using them to facilitate the early detection, diagnosis, and treatment of sepsis.

## 5. Conclusions

In summary, our study provides evidence that haematological parameters could be useful in predicting patient outcomes in the absence of positive blood cultures. We explored the use of newer features of automated haematology analysers to assess cell populations related to the activation states of neutrophils, lymphocytes, and monocytes, and found promising results in stratifying sepsis patients. However, further studies with larger sample sizes are necessary to validate our findings and to establish the clinical utility of haematological parameters in predicting patient outcomes in the ICU. 

## Figures and Tables

**Table 1:** Demographic profile and association of laboratory data with different studied participants.

Parameters	Sepsis (*n* = 30)	Control Arm		*p* Value
		Non-Sepsis (*n* = 23)	Healthy (*n* = 30)	
Age(year)	58 (13)	49 (16)	34 (13)	
Gender				
Female	14 (46.7%)	9 (39.1%)	10 (33.3%)	
Male	16 (53.3%)	14 (60.9%)	20 (66.7%)	
Blood Culture				
Positive	7 (23.3%)	0	NA	
Negative	23 (76.7%)	23 (100%)	NA	
Clinical Outcome				
Death	10 (33.3%)	0	NA	
Discharged	20 (66.7%)	23 (100%)	NA	
Laboratory Results				
SOFA	7.10 (3.02)	0.96 (0.21)	0.00 (0.00)	<0.001
CRP (mg/L)	142.60 (47.85)	75.84 (57.69)	0.50 (0.00)	<0.001
PCT (ng/mL)	26.45 (36.63)	0.25 (0.11)	0.10 (0.00)	<0.001
IL-6 (pg/mL)	59.02 (42.07)	28.34 (37.17)	1.56 (0.00)	<0.001
TWBC (10^3^/μL)	13.82 (5.50)	13.00 (4.99)	7.05 (1.87)	<0.001
NEUT# (10^3^/μL)	12.26 (5.71)	10.88 (4.56)	3.85 (1.37)	<0.001
NEUT-GI (SI)	150.80 (5.97)	153.33 (4.98)	149.13 (2.80)	0.008
NEUT-RI (FI)	56.49 (10.36)	52.20 (6.15)	45.61 (1.89)	<0.001
IG# (10^3^/μL)	0.47 (0.77)	0.17 (0.22)	0.05 (0.03)	0.004
MONO# (10^3^/μL)	0.73 (0.31)	0.85 (0.53)	0.53 (0.17)	0.004
LYMPH# (10^3^/μL)	1.04 (0.52)	1.18 (0.54)	2.30 (0.57)	<0.001
RE-LYMP (10^3^/μL)	0.11 (0.11)	0.06 (0.05)	0.04 (0.03)	0.001
AS-LYMP (cells/μL)	0.05 (0.07)	0.02 (0.02)	0.01 (0.02)	0.014
PLT# (10^3^/μL)	197.77 (109.92)	282.65 (123.12)	292.83 (78.68)	0.001
IPF %	5.69 (4.32)	3.27 (2.06)	4.09 (2.45)	0.021
Delta-He (pg)	0.14 (3.62)	2.01 (2.19)	2.85 (1.73)	0.001
NE-WX (ch)	338.57 (33.96)	314.65 (17.00)	314.77 (10.94)	<0.001
NE-WY (ch)	750.87 (175.04)	673.78 (91.44)	601.30 (24.55)	<0.001
NE-WZ (ch)	659.67 (48.42)	606.22 (26.48)	634.93 (27.51)	<0.001
RE-MONO# (10^3^/μL)	0.015 (0.030)	0.003 (0.007)	0.004 (0.005)	0.037

All the variables were analysed by one-way ANOVA. *p* < 0.05 was statistically significant. SD, standard deviation; df, degree of freedom; SOFA, Sequential Organ Failure Assessment; CRP, C-reactive protein; PCT, procalcitonin; IL-6, interleukin 6; TWBC, total white blood cell count; NEUT#, neutrophil count; NEUT-GI, neutrophil granularity intensity; NEUT-RI, neutrophil reactivity intensity; IG#, immature granulocyte count; MONO, monocyte count; LYMPH#, lymphocyte count; RE-LYMPH, reactive lymphocyte; AS-LYMPH, antibody synthesizing lymphocyte; PLT, platelet count; IPF%, immature platelet fraction in percentage; Delta-He, delta haemoglobin; NE-WX, the width of the lateral scattered light distribution width; NE-WY, the width of the fluorescent light distribution of the neutrophil scattergram; NE-WZ, the forward scattered light distribution; RE-MONO, reactive monocyte; ICIS, intensive care infection score.

## Data Availability

The data collected and analysed during this study are available from the corresponding author upon reasonable request.
